# Atypical Genetic Basis of Pyrazinamide Resistance in Monoresistant Mycobacterium tuberculosis

**DOI:** 10.1128/AAC.01916-20

**Published:** 2021-05-18

**Authors:** Samuel J. Modlin, Tyler Marbach, Jim Werngren, Mikael Mansjö, Sven E. Hoffner, Faramarz Valafar

**Affiliations:** a Laboratory for Pathogenesis of Clinical Drug Resistance and Persistence, School of Public Health, San Diego State University, San Diego, California, USA; b Department of Microbiology, Public Health Agency of Sweden, Solna, Sweden; c Department of Global Public Health, Karolinska institute, Stockholm, Sweden

**Keywords:** pyrazinamide, pyrazinamide resistance, *clpC1*, mode of action, antimicrobial resistance, low-level resistance, monoresistance, *Mycobacterium tuberculosis*, antibiotic resistance, *pncA*

## Abstract

Pyrazinamide (PZA) is a widely used antitubercular chemotherapeutic. Typically, PZA resistance (PZA-R) emerges in Mycobacterium tuberculosis strains with existing resistance to isoniazid and rifampin (i.e., multidrug resistance [MDR]) and is conferred by loss-of-function *pncA* mutations that inhibit conversion to its active form, pyrazinoic acid (POA). PZA-R departing from this canonical scenario is poorly understood. Here, we genotyped *pncA* and purported alternative PZA-R genes (*panD*, *rpsA*, and *clpC1*) with long-read sequencing of 19 phenotypically PZA-monoresistant isolates collected in Sweden and compared their phylogenetic and genomic characteristics to a large set of MDR PZA-R (MDR_PZA-R_) isolates. We report the first association of ClpC1 mutations with PZA-R in clinical isolates, in the ClpC1 promoter (*clpC1p*_−138_) and the N terminus of ClpC1 (ClpC1_Val63Ala_). Mutations have emerged in both these regions under POA selection *in vitro*, and the N-terminal region of ClpC1 has been implicated further, through its POA-dependent efficacy in PanD proteolysis. ClpC1_Val63Ala_ mutants spanned 4 Indo-Oceanic sublineages. Indo-Oceanic isolates invariably harbored ClpC1_Val63Ala_ and were starkly overrepresented (odds ratio [OR] = 22.2, *P* < 0.00001) among PZA-monoresistant isolates (11/19) compared to MDR_PZA-R_ isolates (5/80). The genetic basis of Indo-Oceanic isolates’ overrepresentation in PZA-monoresistant tuberculosis (TB) remains undetermined, but substantial circumstantial evidence suggests that ClpC1_Val63Ala_ confers low-level PZA resistance. Our findings highlight ClpC1 as potentially clinically relevant for PZA-R and reinforce the importance of genetic background in the trajectory of resistance development.

## INTRODUCTION

The primary mechanism of resistance to pyrazinamide (PZA) is inactivation or diminished expression of pyrazinamidase/nicotinamidase (PZase) (1), which converts PZA to its active form, pyrazinoic acid (POA). Dozens of unique mutations in the gene encoding PZase, *pncA*, reportedly confer resistance (2) through this mechanism, though not all appear in clinical isolates (3). A more limited number of *pncA* mutations have been described not to influence the susceptibility to PZA (3). The mode of POA action is less clear. Several models have been proposed for the mode of action of and alternative mechanisms of Mycobacterium tuberculosis POA/PZA (1). These include disruption of ribosomal salvage via *trans*-translation inhibition (4) due to POA binding of the 30S ribosomal subunit S1 (RpsA) and disrupting coenzyme A (CoA) synthesis (5) by inhibiting the rate-limiting enzyme of the pathway (6), l-aspartate decarboxylase (PanD) (7). Two groups have identified a connection between POA resistance and ClpC1 (8, 9), an AAA+ ATPase that acts as the chaperone for the cytoplasmic Clp protease (10). Recent work (11) proposed a mechanism for ClpC1-mediated POA resistance based on the finding that altered Clp protease degradation of PanD functions as part of mechanism of POA action and that mutation in ClpC1 of the Clp complex (Clp protease plus ClpC1) mitigates POA-driven efficiency of PanD proteolysis. Particularly in cases where *pncA* mutations are absent, the targets mediating resistance against these proposed modes of action are candidates for conferring phenotypic resistance.

A recent analysis of 26 isolates from Sweden with PZA-resistant (PZA-R) but wild-type (WT) *pncA* was conducted to understand the nature of this noncanonical genetic basis of PZA-R (12). That study was able to attribute resistance to false resistance in 8/26 isolates through replicate drug-susceptibility testing (DST) at two concentrations and resistance in another seven isolates to heteroresistance by *pncA* genotyping following drug pressure in a Bactec mycobacterial growth indicator tube (MGIT) PZA test. To potentially explain the 11 remaining unexplained isolates, the authors of that study genotyped proposed alternative PZA resistance genes (*rpsA* and *panD*) with Ion Torrent sequencing. All 11 appeared to be explained by either mixed populations with naturally PZA-R Mycobacterium avium (*n* = 3), *pncA* mutation not captured by their primer (*n* = 1), or alternative mechanisms involving mutation in *panD* (*n* = 4) or *rpsA* (*n* = 3). However, they found that 17/400 PZA-S isolates harbored nonsynonymous *rpsA* mutations, including five with the same mutation seen in two of their three PZA-R *rpsA* mutants (12).

Here, we used long-read sequencing data to genotype 19 PZA-monoresistant isolates from Sweden, including nine of the WT-*pncA* PZA-R isolates described above. We contrast the genetic basis of PZA resistance in these isolates with that of a large set of multidrug-resistant (MDR_PZA-R_) isolates isolated in Sweden during a similar time period to identify aspects of PZA resistance that distinguish PZA monoresistance from MDR_PZA-R_. We analyzed *pncA* and genes suggested or shown to mediate alternative resistance mechanisms (*panD*, *rpsA*, and *clpC1*), focusing on how the prevalence of mutations in these genes differs between PZA-monoresistant isolates and MDR_PZA-R_ isolates.

## RESULTS AND DISCUSSION

### PZA-monoresistant clinical isolates.

Isolates were selected from samples collected in Swedish clinical tuberculosis (TB) labs (from patients in Sweden who were most likely infected primarily in Asia and Africa) and processed at the Public Health Agency of Sweden between 2000 and 2015 on the basis of PZA-R in initial phenotypic drug susceptibility testing (DST) and phenotypic susceptibility to isoniazid, rifampin, ethambutol, and amikacin. Genotypic DST corroborated the phenotypic results and implied susceptibility to fluoroquinolones and second-line injectable drugs, with only established lineage markers observed (Table 1). PZA-monoresistant isolates span three of the four major M. tuberculosis lineages and multiple sublineages within the Indo-Oceanic and Euro-American lineages (Table 1).

**TABLE 1:** Lineage and genotypic resistance to first- and second-line drugs for PZA-monoresistant clinical isolates^*a*^

Isolate	Phylogenetic typing		Mutation^*b*^					
			INH		RIF	FQ		SLIDs
	Lineage^*c*^	Spoligotyping^*d*^	*katG*	*fabG1p*	*rpoB*	*gyrA*	*gyrB*	*rrs*
SEA12126	IO	EAI5 (SIT 1082)	Arg463Leu	WT	Synonymous	Glu21Gln, Ser95Thr, Ala384Val, Gly668Asp	Ala130Ser, Met291Ile	WT
SEA12334	IO	EAI6_BGD1 (SIT 43)	Arg463Leu	WT	Synonymous	Glu21Gln, Ser95Thr, Ala384Val, Gly668Asp	Met291Ile	WT
SEA13111	IO	EAI3-IND (SIT 414)	Arg463Leu	WT	Synonymous	Glu21Gln, Ser95Thr, Ala384Val, Gly668Asp	Met291Ile	WT
SEA12202	IO	NA	Arg463Leu	WT	Synonymous	Glu21Gln, Ser95Thr, Ala384Val, Gly668Asp	Met291Ile	WT
SEA00042	EAM	NA	WT	WT	Synonymous	Glu21Gln, Ser95Thr, Gly668Asp	WT	WT
SEA06535	EAM	U (SIT 1142)	WT	WT	Synonymous	Glu21Gln, Ser95Thr, Gly668Asp	WT	WT
SEA08151	IO	EAI1_SOM (SIT 48)	Arg463Leu	WT	Synonymous	Glu21Gln, Ser95Thr, Ala384Val, Gly668Asp	Met291Ile	G883A
SEA08162	EAM	LAM4 (SIT 1530)	WT	WT	Glu21Gln, Ser95Thr, Gly247Ser, Gly668Asp	WT	WT
SEA10007	IO	EAI1_SOM (SIT 48)	Arg463Leu	WT	Synonymous	Glu21Gln, Ser95Thr, Ala384Val, Gly668Asp	Met291Ile	G883A
SEA11278	Ethiopian	SIT 910	Arg463Leu	WT	Synonymous	Glu21Gln, Ser95Thr, Gly668Asp	WT	WT
SEA14117	EAS	Beijing-like (SIT 269)	Arg463Leu	WT	Synonymous	Glu21Gln, Ser95Thr, Gly668Asp	WT	WT
SEA14318	IO	EAI5 (SIT 138)	Arg463Leu	WT	Synonymous	Ser95Thr, Glu214Asp, Ala384Val, Gly668Asp	Met291Ile	WT
SEA15209	*M. canettii*	NA	Arg463Leu	C-183G	Synonymous	Glu21Gln, Ser95Thr, Asp504Glu, Gly668Asp, -ATCAGGCTC2518	WT	T6G
SEA10470	EAM	H3 (SIT 50)	WT	WT	Synonymous	Glu21Gln, Ser95Thr, Gly668Asp	WT	WT
SEA09167	EAM	LAM11_ZWE (SIT 59)	WT	WT	WT	Glu21Gln, Ser95Thr, Gly668Asp	Val301Leu	WT
SEA14333	IO	EAI1-SOM (SIT 735)	Arg463Leu	WT	Synonymous	Glu21Gln, Ser95Thr, Ala384Val, Gly668Asp	Met291Ile	G1454C
SEA15228	IO	EAI1-SOM (SIT 48)	Arg463Leu	WT	Synonymous	Glu21Gln, Ser95Thr, Ala384Val, Gly668Asp	Met291Ile	WT
SEA15229	IO	EAI3-IND (SIT 11)	Arg463Leu	WT	Synonymous	Glu21Gln, Ser95Thr, Ala384Val, Gly668Asp	Met291Ile	WT
SEA15230	IO	EAI5 (SIT 299)	Arg463Leu	WT	Synonymous	Glu21Gln, Ser95Thr, Ala384Val, Gly668Asp	Met291Ile	WT

a Isolates were collected from TB patients in Sweden between 2000 and 2015.

b Mutations in known resistance genes. Genes with a wild-type allele (WT) are those matching reference and virulent type strain H37Rv. INH, isoniazid; RIF, rifampin; FQ, fluoroquinolones; SLIDs, second-line injectable drugs.

c Defined according to large sequence polymorphism classification (34). IO, Indo-Oceanic; EAM, Euro-American; EAS, East Asian.

d NA, not available (spoligotyping data unavailable).

With a single exception, PacBio sequencing calls were concordant with initial Sanger genotyping of *pncA* and with genotyping of alternative resistance targets by Sanger and Ion Torrent sequencing. The sole exception was an isolate (SEA10470) (Table 2) initially identified as WT/Ser65Ser (a minor population of Ser65Ser) but had a large deletion spanning the beginning of *pncA* and its upstream region in the PacBio data (Table 2). In their recent work (which included SEA10470), upon observing that their *pncA* primer for Sanger sequencing from the MGIT PZA tube did not return anything to sequence, Werngren and colleagues sequenced the PZA-containing MGIT-derived sample on Ion Torrent (12) and discovered the same large deletion that we found in single-molecule real-time (SMRT) sequencing data following several weeks of growth in Löwenstein-Jensen (LJ) medium, implying that WT-*pncA* was ultimately outcompeted by the *pncA* deletion in both the presence (MGIT PZA tube) and absence of PZA (growth in LJ medium preceding SMRT sequencing). Thus, we conclude SEA10470 is a heteroresistant *pncA* mutant with limited or no fitness cost in LJ medium.

**TABLE 2:** Genotypic and phenotypic profiles of 19 monoresistant PZA samples isolated in Sweden

Isolate	Lineage^*a*^	PZA DST result		*pncA*	Resistance conferring^*b*^	Genotype			Resistance explanation^*c*^
		Initial	MGIT (100/200 mg/liter)			*panD*	*rpsA*	*clpC1*	
SEA12126	IO	R	S/S	WT	WT	Val260Ile	Val63Ala	DST flipped
SEA12334	IO	R	S/S	WT	WT	WT	Val63Ala	DST flipped
SEA13111	IO	R	S/S	WT	WT	WT	Val63Ala	DST flipped
SEA12202	IO	R	R/R	Leu172Pro	Y	WT	WT	Val63Ala	*pncA* mediated
SEA00042	EAM	R	R/R	His51Gln	Y	WT	WT	WT	*pncA* mediated
SEA06535	EAM	R	R/R	Arg123Pro	Y	WT	WT	WT	*pncA* mediated
SEA08151	IO	R	R/R	-A416	Y^*d*^	WT	WT	Val63Ala	*pncA* mediated
SEA08162	EAM	R	R/R	Leu172Pro	Y	WT	WT	Asn806Asn	*pncA* mediated
SEA10007	IO	R	R/R	-A416	Y^*d*^	WT	WT	Val63Ala	*pncA* mediated
SEA12178	Ethiopian	R	R/R	Cys138Tyr	Y	Asp133Ala	Thr459Pro	WT	*pncA* mediated
SEA14117	EAS	R	R/R	Ile90Ser	Y	WT	Arg212Arg	Tyr389Tyr, promoter: −C138	*pncA* mediated
SEA14318	IO	R	R/R	DVVG129-132G	Y^*e*^	WT	WT	Thr241Thr, Val63Ala	*pncA* mediated
SEA15209	*M. canettii*	R	R/R	Ala46Ala	NA	Met117Thr, Ala13Ala	Thr5Ala, Pro9Pro, Thr210Ala, Glu457Glu	promoter: +A-170, A-177G, 49 synonymous mutations	Intrinsic PZA resistance
SEA10470	EAM	R	R/R	deleted nucleotides 264–158	Y^*f*^	WT	WT	WT	Heteroresistance^*g*^
SEA09167	EAM	R	R/R	WT	Ile115Thr	WT	Asn806Asn	Alternative mechanism
SEA14333	IO	R	R/R	WT	WT	Ile55Val, Val260Ile	Val63Ala	Alternative mechanism
SEA15228	IO	R	R/R	WT	WT	Val260Ile	Val63Ala	Alternative mechanism
SEA15229	IO	R	R/S	WT	WT	WT	Val63Ala	Alternative mechanism
SEA15230	IO	R	R/R	WT	Ile49Val	WT	Val63Ala	Alternative mechanism

a IO, Indo-Oceanic (lineage 1); EAM, Euro-American (lineage 2); EAS, East-Asian (lineage 4). Ethiopian is lineage 7.

b Resistance mutations described by Yadon et al. (2) and/or Ramirez-Busby and Valafar (3). NA, not applicable (M. canetti is intrinsically PZA resistant).

c “DST flipped” indicates that no genotyping of MGIT from PZA tube was performed.

d Other frameshifts in this region have been reported to confer resistance (3).

e Other deletions affecting these residues have been reported to confer resistance (3).

f Certainly would disrupt PncA expression and ostensibly confer resistance.

g Complex case of heteroresistance described in the text.

### *panD*, *rpsA*, and *clpC1* mutations in PZA-monoresistant isolates with wild-type *pncA*.

To identify the genetic basis of our PZA-monoresistant isolates, we first checked for *pncA* mutations. PncA alleles for all 10 *pncA* mutants had been reported previously for clinical isolates (Table 3), substantiating their role in conferring PZA-R to these isolates. After accounting for isolates with *pncA* mutations (Table 3), PZA-R in nine WT-*pncA* isolates remained unexplained. One isolate is Mycobacterium canettii and is therefore naturally PZA-R (13), leaving 8 PZA-R WT-*pncA* M. tuberculosis isolates. This prevalence of noncanonical PZA resistance among monoresistant M. tuberculosis isolates (8/18) is remarkably higher (*P < *0.00001, odds ratio = 32.2, 95% confidence interval [CI] = 35.47 to 351, two-tailed Fisher’s exact test) than in MDR_PZA-R_ Swedish isolates (2/80), suggesting that the nature of PZA monoresistance is different from that of PZA resistance in MDR isolates. All eight had at least one nonsynonymous mutation in *panD*, *rpsA*, or *clpC1.* Three of the eight were susceptible upon repeated phenotypic DST, and one exhibited low-level resistance (resistant at 100 μg/ml but not 200 μg/ml). All four isolates that remained unexplained, with neither PncA mutations nor inconsistent phenotyping, had multiple mutations in alternative PZA resistance genes. Two had nonsynonymous mutations in *panD* and the other two in *rpsA* (Table 2), and all had the missense mutation ClpC1_Val63Ala_. However, the *rpsA* mutations also occurred in many Swedish isolates susceptible to PZA (12), suggesting either that these mutations confer low-level resistance around the critical concentration or that other genetic elements are at play.

**TABLE 3:** Sublineage diversity of PZA-monoresistant *clpC1*_Val63Ala_ mutants within the Indo-Oceanic lineage^*a*^

Sublineage	Isolate	Spoligotype
1.1.1	SEA12126	EAI5 (SIT 1082)
SEA15228	EAI1 SOM (SIT 48)
SEA14333	EAI1 SOM (SIT 735)
1.1.2	SEA15230	EAI5 (SIT 299)
SEA15229	EAI3 IND (SIT 11)
SEA13111	EAI3 IND (SIT 414)
1.2.1	SEA12334	EAI6 BGD1 (SIT 43)
SEA12202	NA
1.2.2	SEA10007	EAI1_SOM (SIT 48)
SEA08151	EAI1 SOM (SIT 48)
SEA14318	EAI5 (SIT 138)

a Sublineage membership was determined using the lineage-specific SNPs described by Coll et al. (19). NA, not available (spoligotyping data are unavailable for this isolate).

### Novel mutations implicate regions mediating PanD proteolysis by ClpC1.

Recent work demonstrated that POA increases PanD proteolysis by the Clp complex and proposed this as its mode of action (11). This proteolysis requires a C-terminal degradation tag (PanD_127–139_) for recognition by the N terminus of ClpC1. While PZA-R *clpC1* mutants have not been previously reported among clinical isolates, they have been isolated following selection under PZA (14) and POA (9) pressure *in vitro* and POA pressure in an *in vivo* mouse model (8). Here, we report the first account of *clpC1* mutations in PZA-R clinical isolates with WT-*pncA* and a *panD* mutation (*panD*_Asp133Ala_) in its C-terminal recognition tag novel to clinical isolates.

The first c*lpC1* mutation is a deletion in the *clpC1* promoter (*clpC1p*), 138 bp upstream of its start codon (*clpC1p*_−C138_). The isolate harboring *clpC1p*_−C138_ (SEA14117) (Table 2) has a *pncA* mutation (*pncA*_Ile90Ser_) previously reported in PZA-R clinical isolates. However, *pncA*_Ile90Ser_ is rare and—unlike most clinically impactful *pncA* mutations (2)*—*is not enriched following selection under PZA pressure *in vitro* or in mice, and neither were any other codon 90 *pncA* mutations. While the possibility that *pncA*_Ile90Ser_ accounts fully for PZA-R in SEA14117 cannot be dismissed, the irreproducibility of *pncA*_Ile90Ser_ conferring resistance on its own *in vitro* leaves open the possibility that *clpC1p*_−C138_ may contribute to resistance either alternatively to *pncA*_Ile90Ser_ or perhaps additively. While *clpC1p*_−C138_ has not been described previously, other *clpC1* promoter mutations have emerged under POA selection pressure *in vitro* (9), suggesting that *clpC1* promoter mutations can confer POA resistance. Viewing this observation through the model of Clp proteolysis of PanD as the POA mode of action (11), *clpC1p* mutations could confer PZA/POA resistance by weakening the promoter, reducing ClpC1 expression and, in turn, Clp-mediated PanD degradation.

The second *clpC1* mutant was a missense mutation in the ClpC1 N terminus, Val63Ala (*clpC1*_Val63Ala_). Eleven of 19 PZA-monoresistant isolates were *clpC1*_Val63Ala_ mutants, exclusively and invariably of the Indo-Oceanic lineage. These 11 isolates span four Indo-Oceanic sublineages (each with multiple isolates) and comprise seven distinct sublineage-spoligotype combinations (Table 3). Thus, PZA-monoresistant *clpC1*_Val63Ala_ mutants are neither a clonal expansion nor monophyletic. Rather, they are spread throughout the Indo-Oceanic lineage. The effects of the variants *clpC1*_Val63Ala_ and *panD*_Asp133Ala_ converge on the interaction between the ClpC1 N-terminal and PanD_127-139_ C-terminal degradation tags (9). Though it is only speculation at present, the functional effects implied by these mutations’ location fits well into the mode of POA action proposed recently by Gopal and colleagues (11).

Indo-Oceanic strains in this study (11/19), which selected isolates on the basis of PZA monoresistance, were far more prevalent (*P < *0.000001, odds ratio = 22.2, 95% CI = 6.05 to 121, two-tailed Fisher’s exact test) than PZA-resistant Indo-Oceanic isolates from Sweden in a study (35) (5/80) in which isolates were selected on the basis of MDR phenotype. The frequency of ClpC1 mutants that we observed here stands in contrast to a recent study of 269 PZA-resistant isolates in China where no isolates harbored a nonsynonymous ClpC1 mutation (16). However, that study had mostly Beijing lineage isolates (lineage was not indicated for the remaining isolates). This overrepresentation of Indo-Oceanic isolates in monoresistant PZA-R strains relative to MDR_PZA-R_ strains has been observed previously in the United States (17) and Iran (18), suggesting that it is a phenomenon not isolated to samples derived from patients in Sweden. This repeatedly observed trend of Indo-Oceanic isolates developing PZA monoresistance, when considered alongside the mechanistic plausibility of ClpC1 N-terminal mutations affecting POA resistance (8, 9, 11, 14) and the vacillating of these ClpC1 mutants around the resistance threshold (Table 2), suggests that *clpC1*_Val63Ala_ may confer low-level PZA resistance to Indo-Oceanic isolates. The missense mutation *clpC1*_Val63Ala_ is specific to and invariably present in Indo-Oceanic isolates (15, 19) and thus would represent a lineage-specific resistance phenotype akin to the intrinsic PZA resistance of the ancestral mycobacteria *M. canettii* (13) and Mycobacterium bovis (1), but at a lower level. However, confirmatory mutagenesis is needed to verify this possibility.

**FIG 1:**
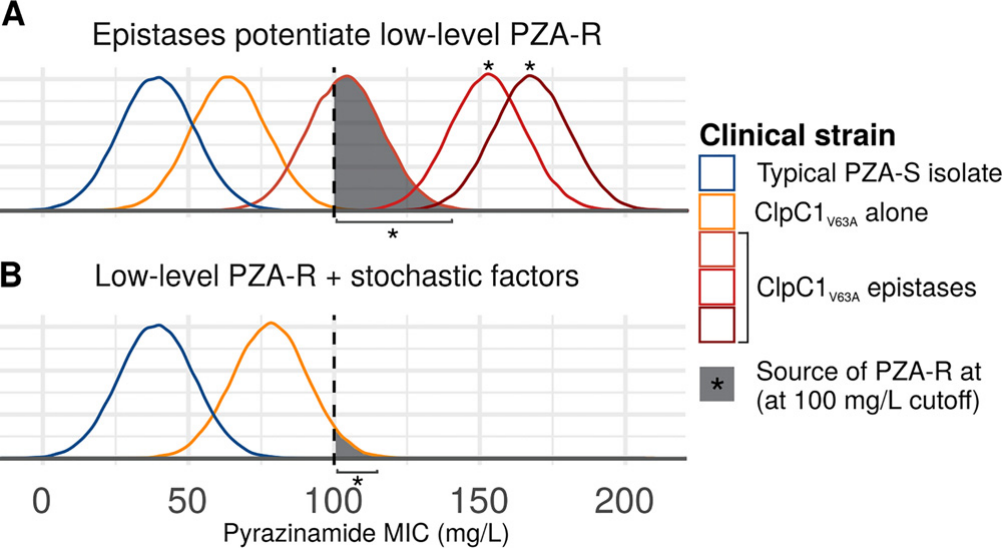
Phenomena potentially underlying low-level PZA-R monoresistance conferred by *clpC1*_Val63Ala_. Curves depict theoretical probability density functions of MICs obtained for a given isolate. (A) Epistasis. In the epistatic scenario, *clpC1*_Val63Ala_ alone confers low-level resistance, invariably below the 100-mg/liter critical concentration, but some isolates (reds) harbor additional mutations that confer higher-level PZA-R through additive or synergistic interaction with *clpC1*_Val63Ala_. (B) Near-critical concentration MIC. In this scenario, low-level PZA resistance conferred by *clpC1*_Val63Ala_ alone sometimes exceeds the 100-mg/liter cutoff, due to nongenetic factors contributing to MIC variance (30, 31), such as inoculum size (32), growth medium, or interlaboratory variation (33). The phenomena depicted in panels A and B are not mutually exclusive and could both be at work.

How might one reconcile the proposed low-level PZA resistance conferred by the *clpC1*_Val63Ala_ allele with the observation that Indo-Oceanic isolates often register as phenotypically susceptible to PZA? It is important to consider that the Bactec MGIT 960 critical concentration (100 μg/ml) has been calibrated to detect *pncA-*conferred resistance, which is severalfold higher than the 2- to 8-fold MIC increases observed for POA-resistant mutants harboring *panD* and *clpC1* mutations (8, 9). This could explain the variable and discrepant phenotypic results we observe in Indo-Oceanic isolates upon replicating DST (Table 2; Fig. 1). This scenario has been reported for PZA/POA-resistant *panD* mutants isolated following selection under POA pressure; despite a 2- to 8-fold MIC increase for G139* *panD* mutants relative to the wild type, seven of eight clinical isolates that Gopal and colleagues found with the mutation were classified as susceptible to PZA (8). Similarly, *rpsA*_Δ438Ala_ and *rpsA*_Asp123Ala_ mutations were recently shown to confer 2-fold increases in MIC relative to H37Rv (20). Low-level resistance conferred by the *clpC1*_Val63Ala_ allele would simultaneously explain the prevalence of Indo-Oceanic lineage and WT-PncA PZA-R among PZA-monoresistant isolates (17, 18) as well as the variable phenotypic results for these isolates. Epistasis (Fig. 1A) and resistance near the critical concentration vacillating above it due to nongenetic factors (Fig. 1B) could also produce the bias toward Indo-Oceanic lineage isolates and inconsistent DST results among monoresistant PZA isolates resulting from *clpC1*_Val63Ala_. In either case, together, these considerations suggest *clpC1*_Val63Ala_ low-level resistance, though confirmation is required. Future work with a larger set of PZA-monoresistant and MDR_PZA-R_ isolates and susceptibility testing across a range of lower MICs would more precisely delineate the role of *clpC1*_Val63Ala_ in PZA resistance.

### Concluding remarks.

By analyzing monoresistant PZA isolates collected in Sweden, we found that monoresistant isolates have mutational profiles distinct from those of MDR_PZA-R_ isolates and identified *clpC1*_Val63Ala_ as a potential low-level resistance marker common to Indo-Oceanic isolates. Despite this mutation’s being common to Indo-Oceanic isolates (19) and POA/PZA-resistant ClpC1 mutants isolated from *in vitro* screens (8, 9), this is, to our knowledge, the first report of PZA-resistant ClpC1 mutants in clinical isolates. Further study of the hypothesized link between *clpC1*_Val63Ala_ and low-level resistance warrants mechanistic study, as does evaluation of its effect on PZA/POA resistance *in vitro* and in the context of infection. The functional effects implicated by *clpC1*_Val63Ala_ and *panD*_Asp133Ala_ converge on the interaction between the ClpC1 N-terminal and PanD_127-139_ C-terminal degradation tags (9), consistent with the emerging model of POA mode of action where PanD degradation by Clp proteolysis is enhanced by POA (11). Functional studies assessing the effect of these two mutations on PanD degradation natively and in the presence of POA would be of great interest. More broadly, the bias of PZA monoresistance toward Indo-Oceanic isolates underlines the relevance of genetic background in evolution of resistance (21–23), a factor that is often precluded from analysis in molecular epidemiology and genome-wide association studies on the assumption that they do not influence resistance. Our findings suggest otherwise.

## MATERIALS AND METHODS

### Isolate selection.

PZA-monoresistant isolates were selected from TB patients seen in Sweden between 2003 and 2015, irrespective of lineage, TB presentation, or geographic origin. The geographic origin of the 19 TB strains isolated in Sweden was typical of the general TB epidemiology in Sweden, with roughly half of the patients coming from Africa, a fifth from Asia, and the rest from Sweden or other parts of the globe (24).

### Culturing and DNA extraction.

M. tuberculosis samples were prepared and extracted at the World Health Organization Supranational Reference Laboratory in Stockholm, Sweden. Isolates growing on LJ medium were received from clinical TB labs and subsequently Sanger sequenced. The genotypic DST result (*pncA*-Sanger) was then compared to the phenotypic DST result (PZA Bactec 460 until 2008, MGIT 960 thereafter) from the clinical lab, and discordant cases were retested in PZA MGIT. In cases of verified PZA resistance, Sanger sequencing was repeated on the growth from the PZA MGIT tube. Samples still lacking a *pncA* mutation were then subjected to whole-genome sequencing (WGS). Preparation and extraction were performed as previously described (12).

### SMRT sequencing.

DNA sequencing was performed at the Institute for Genomic Medicine at the University of California, San Diego, CA. DNA libraries for PacBio (Pacific Biosciences, Menlo Park, CA) were prepared using PacBio’s DNA template prep kit with no follow-up PCR amplification. Briefly, sheared DNA was end repaired, and hairpin adapters were ligated using T4 DNA ligase. Incompletely formed SMRTbell templates were degraded with a combination of exonuclease III and exonuclease VII. The resulting DNA templates were purified using solid-phase reversible-immobilization (SPRI) magnetic beads (AMPure; Agencourt Bioscience, Beverly, MA) and annealed to a 2-fold molar excess of a sequencing primer that specifically bound to the single-stranded loop region of the hairpin adapters. SMRTbell templates were subjected to standard SMRT sequencing using an engineered phi29 DNA polymerase on the PacBio RS II system according to the manufacturer’s protocol.

### Sanger and Ion Torrent sequencing.

DNA sequencing with Sanger and Ion Torrent was carried out at World Health Organization Supranational Reference Laboratory in Stockholm, Sweden, as previously described (12).

### Genotyping from SMRT sequencing data.

Raw Pacific Biosciences SMRT sequencing reads were aligned to the reference genome of the M. tuberculosis virulent type strain H37Rv reference strain (GenBank accession number NC_000962.3) using BLASR (25) (v1.3) with default parameters. PBHoover (26) (https://gitlab.com/LPCDRP/pbhoover) corrected aligned reads and called variants based on a maximum-likelihood criterion. VCF formatted files were further annotated with Variant Effect Predictor (27) (VEP) (v87) to determine the consequence of each variant. Variants within or proximally upstream of *clpC1*, *panD*, *rpsA*, and *pncA* were screened for using a custom python script (https://gitlab.com/LPCDRP/drug-resistance/-/blob/master/src/known-resistance-association.py).

### Lineage determination and spoligotyping.

Lineage was determined by *in silico* mycobacterial interspersed repetitive unit (MIRU) typing and spoligotyping with MiruHero (https://gitlab.com/LPCDRP/miru-hero). Spoligotyping was also carried out in Stockholm, Sweden, as described previously (28). Sublineage membership for Indo-Oceanic isolates was determined by the lineage-specific single nucleotide polymorphisms (SNPs) defined by Coll and colleagues (19).

### Drug susceptibility testing.

For cases with discordance between initial Sanger sequencing of *pncA* and the PZA Bactec 460/MGIT result from the clinical lab, PZA DST was performed in the Bactec MGIT system (Becton Dickinson) according to instructions from the manufacturer. The DST inoculum was prepared from bacterial growth on Löwenstein-Jensen egg medium at 37°C. Briefly, two 1-μl loops of bacteria were suspended in 3 ml phosphate-buffered saline (PBS) in a small glass tube with glass beads. The bacterial suspension was homogenized using vortexing or an ultrasound water bath to disperse any clumps. The suspension was then left to sediment for 20 min, and the upper 2 ml was transferred to a new tube and left to sediment for another 15 min. Before inoculation of the MGIT PZA medium culture tubes (pH 5.9), the bacterial suspension was adjusted to a McFarland turbidity of 0.5 and diluted in PBS per the manufacturer’s PZA test protocol.

### Statistical tests.

Two-tailed Fisher’s exact test was used to assess independence and implemented in R statistical programming language (29).

### Data availability.

All code is available on GitLab at https://gitlab.com/LPCDRP/miru-hero.
